# Changes in soil bacterial community diversity following the removal of invasive feral pigs from a Hawaiian tropical montane wet forest

**DOI:** 10.1038/s41598-019-48922-7

**Published:** 2019-10-11

**Authors:** Nathaniel H. Wehr, Kealohanuiopuna M. Kinney, Nhu H. Nguyen, Christian P. Giardina, Creighton M. Litton

**Affiliations:** 1 Department of Natural Resources & Environmental Management, University of Hawai’i at Mānoa, Honolulu, HI 96822 USA; 2 Institute of Pacific Islands Forestry, USDA Forest Service, Hilo, HI 96720 USA; 3 Department of Tropical Plant & Soil Science, University of Hawai’i at Mānoa, Honolulu, HI 96822 USA

**Keywords:** Invasive species, Microbial ecology

## Abstract

Nonnative, invasive feral pigs (*Sus scrofa*) modify habitats by disturbing soils and vegetation, which can alter biogeochemical processes. Soil microbial communities drive nutrient cycling and therefore also play important roles in shaping ecosystem structure and function, but the responses of soil microbes to nonnative ungulate removal remains poorly studied. We examined changes in the soil bacterial community over a ~25 year chronosequence of feral pig removal in tropical montane wet forests on the Island of Hawai’i. We extracted bacterial eDNA from soil samples collected inside and outside of ungulate exclosures along this chronosequence and sequenced the eDNA using the Illumina platform. We found that ungulate removal increased diversity of soil bacteria, with diversity scores positively correlated with time since removal. While functional and phylogenetic diversity were not significantly different between pig present and pig removed soils, soil bulk density, which decreases following the removal of feral pigs, was a useful predictor of dissimilarity among sites and correlated to changes in functional diversity. Additionally, increases in soil porosity, potassium, and calcium were correlated to increases in functional diversity. Finally, sites with greater mean annual temperatures were shown to have higher scores of both functional and phylogenetic diversity. As such, we conclude that feral pigs influence overall bacterial community diversity directly while influencing functional diversity indirectly through alterations to soil structure and nutrients. Comparatively, phylogenetic differences between communities are better explained by mean annual temperature as a climatic predictor of community dissimilarity.

## Introduction

Soil microbes play critical roles in water retention and purification, nutrient cycling, primary production, soil formation and processing, and carbon sequestration, with individual taxa capable of contributing either highly specialized or generalist roles^1^. As scientists have begun to recognize the important roles microbes play in these processes, studies examining the soil microbiome have become increasingly common. As a result, numerous factors including pH, soil water content^2,3^, cation exchange capacity^3^, and soil organic matter content^4^ have been shown to influence the composition, structure, and function of soil microbial communities.

Given that many soil microbes are highly sensitive to these localized environmental factors, the soil microbiome has been associated with both above- and belowground plant and animal communities^1,2,5–7^. For example, microbial biomass and respiration have been shown to increase with increases in plant species richness^8^, and the microbiome in another study was more diverse under native tree canopies than under canopies of non-native plants^9^. Further, the global beta-diversity of grassland plants has been strongly correlated to the beta-diversity of soil microbial communities^10^. The removal of ungulate grazing was also shown to decrease biodiversity in grasslands^11,12^ and reduce microbial activity in a forest ecosystem^6^.

In consideration of the potential associations between ungulate and microbial communities, the effects of specific ungulates, such as invasive feral pigs (*Sus scrofa*), on aboveground environments are often readily visible and can translate into indirect effects on the belowground community of microorganisms^13^. Prior research has shown that feral pigs can influence nearly all aspects of their habitat^14–16^. Resultant changes include decreased diversity and abundance of plants^17,18^, fungi^19^, and wildlife^20,21^ as well as being associated with the increased presence of specific bacteria in watersheds^22–24^. Comparatively, the effects of feral pigs on soils are typically highly variable, due to the narrow, localized effects of their trampling, wallowing, and rooting behaviors. At the level of individual study plots, examinations of feral pig influences on soils have shown mixed results. For example, in Tennessee’s deciduous forests, feral pig activity reduced soil bulk density (SBD)^25^, while SBD increased with feral pig activity in both Spanish alpine habitats^26^ and Hawaiian tropical montane wet forests^27^. Physical alteration of soil organic matter via foraging was also documented in some^28–30^, but not all ecosystems where it has been examined^31^. Moreover, feral pigs also influence soil nutrient cycling and availability, with several studies documenting increased soil nitrogen (N) availability in the presence of feral pigs^26,29,30^.

Feral pigs have also been associated with direct effects on soil microbial communities. Specifically, feral pigs have been shown to affect the soil microbial community through defecation^6^ and soil turbation^32^ resulting in alterations specific to the composition of soil microbial communities^33^. For example, feral pigs have been associated with an increased presence of fecal coliform in watersheds^22^ and have also been shown to increase the presence of specific bacteria, such as enterococci and leptospira, in soil runoff^23,24,34^. Feral pig activity has been directly associated with increased soil microbial activity attributed to potential increases of soil N availability associated with feces and urine^6,35^ and indirectly via increased availability of soil organic matter^36^. Further, the presence of feral pigs has been associated with faster microbial community recovery following rapid unexpected changes in the soil environment, such as the application of fumigants^37^.

In Hawai’i, and throughout much the Pacific Island region, feral pigs serve an important role culturally, but have been associated with many negative alterations to native ecosystems. As a result, land managers primarily utilize exclosure fencing coupled with removal as the primary non-lethal management strategy for feral pigs in Hawai’i^16^. Prior research examining the outcomes of this management strategy have indicated that the removal of feral pigs from Hawaiian tropical montane wet forests increases understory plant species richness and density, increases the cycling and availability of soil N, and decreases SBD and soil aggregation^17,18,27^. In Hawai’i, examinations of microbial changes following feral pig removal have been limited to short-term (<2 years) examinations of individual taxa and did not analyze changes to the entire community^22–24^. Additionally, long-term studies of ungulate removal occurring globally have been largely limited to grassland systems^11,12^. As such, our goal was to examine the effects of feral pig removal on soil microbial communities across a 25-year chronosequence of removal in a forest ecosystem in Hawai’i. We asked the following questions: (1) How does the removal of feral pigs affect community and functional diversity of soil bacteria?; (2) Do these effects change with time since pig removal?; and (3) How do differences in environmental variables (e.g. climatic, floral, and soil variation; Table 1) affect the community and functional diversity of soil bacteria?

**Table 1:** Study site characteristics.

Site	Lat. (°)	Long. (°)	Age (year)	Size (ha)	MAP (mm)^45^	MAT (°C)^45^	Elev. (m)^18^	Soil Series^18^	SBD (g/cm^3^)^27^	VWC (%)^27^	WPS (%)^27^
SP (%)^27^	K (mg/cm^3^)^27^	Ca (mg/cm^3^)^27^	Mg (mg/cm^3^)^27^	GC:L (%)^18^	GC:B (%)^18^	TS (#/m^2^)^18^	SD (#/ha)^18^	BA (m^2^/ha)^18^			
Aku In	−155.234	19.544	2002	117	3,984	15.8	1,143	Eheuiki	0.27 ± 0.05	77.8 ± 1.2	83.3 ± 4.1
Koa In	−155.234	19.486	1994	1,024	3,320	15.8	1,158	Puaulu	0.31 ± 0.11	56.6 ± 5.6	69.4 ± 12
Lava	−155.267	19.511	2004	152	2,997	15.0	1,311	Eheuiki	0.19 ± 0.01	55.8 ± 3.8	68.1 ± 2.8
NLM	−155.272	19.511	2001	223	2,938	14.8	1,341	Eheuiki	0.19 ± 0.01	54.4 ± 8.0	68.4 ± 8.6
Puu	−155.262	19.495	1992	240	2,910	15.0	1,295	Puaulu	0.19 ± 0.03	48.3 ± 4.9	68.1 ± 4.3
Aku Out	−155.239	19.545	—	—	3,903	15.6	1,173	Eheuiki	0.26 ± 0.05	74.5 ± 1.7	78.6 ± 1.8
Koa Out	−155.229	19.490	—	—	3,474	15.9	1,143	Puaulu	0.36 ± 0.02	61.0 ± 2.5	79.2 ± 1.5
Olaa	−155.234	19.504	—	—	3,473	15.8	1,158	Puaulu	0.53 ± 0.05	65.4 ± 4.3	77.3 ± 4.7
PMA	−155.278	19.521	—	—	2,949	14.5	1,372	Eheuiki	0.21 ± 0.06	65.5 ± 6.1	73.4 ± 7.3
89.8 ± 1.8	0.17 ± 0.05	1.86 ± 0.48	0.38 ± 0.08	52.4 ± 5.5	42.2 ± 3.3	9.2 ± 4.4	3,817 ± 645	92.1 ± 12.3			
88.3 ± 4.2	0.11 ± 0.06	1.68 ± 0.17	0.43 ± 0.09	79.0 ± 3.0	13.9 ± 1.5	3.7 ± 1.6	3,556 ± 710	196.2 ± 13.0			
92.7 ± 0.4	0.27 ± 0.06	2.24 ± 0.48	0.44 ± 0.06	76.2 ± 3.5	17.5 ± 2.8	2.6 ± 0.4	2,873 ± 517	121.9 ± 23.3			
92.9 ± 0.6	0.25 ± 0.10	2.32 ± 0.42	0.43 ± 0.07	80.4 ± 6.8	15.1 ± 4.5	1.7 ± 1.0	2,623 ± 599	113.6 ± 25.5			
92.6 ± 1.1	0.22 ± 0.04	2.21 ± 0.64	0.50 ± 0.17	85.4 ± 3.2	9.5 ± 3.5	1.8 ± 1.0	3,215 ± 573	111.9 ± 12.8			
90.1 ± 2.0	0.13 ± 0.04	1.53 ± 0.48	0.31 ± 0.08	62.3 ± 10.9	25.3 ± 7.0	2.1 ± 1.4	2,527 ± 112	94.2 ± 13.7			
86.2 ± 0.6	0.10 ± 0.00	1.67 ± 0.29	0.40 ± 0.06	60.9 ± 10.9	15.9 ± 3.4	0.5 ± 0.6	4,052 ± 1,280	77.3 ± 15.4			
79.9 ± 1.9	0.05 ± 0.00	1.56 ± 0.21	0.37 ± 0.08	59.2 ± 10.8	11.4 ± 2.8	0.1 ± 0.2	3,856 ± 360	79.6 ± 12.5			
91.9 ± 2.1	0.19 ± 0.05	1.95 ± 0.60	0.34 ± 0.05	53.9 ± 12.9	18.1 ± 2.0	0.8 ± 1.2	3,014 ± 396	106.9 ± 4.0			

Study site characteristics in the feral pig present and feral pig removal sites in the Ola’a Unit of Hawai’i Volcanoes National Park and Pu’u Maka’ala Natural Area Reserve. Abbreviations: Lat. (latitude), Long. (longitude), Age (year pigs were removed from the unit), Size (area of removal unit), MAP (mean annual precipitation), MAT (mean annual temperature), Elev. (elevation), SBD (soil bulk density), VWC (volumetric water content), WPS (water-filled pore space), SP (soil porosity), K (potassium), Ca (calcium), Mg (magnesium), GC:L (ground covered by litter), GC:B (ground covered by bryophytes), TS (count of terrestrial seedlings), SD (stand density of large trees, small trees and shrubs, and tree ferns), and BA (sum of basal area of large trees, small trees and shrubs, and tree ferns). Large trees were defined as those with dbh >20 cm. Small trees and shrubs were those with dbh <20 cm and >1 cm. Errors represent standard deviation.

Given the potential for direct effects of feral pigs on the bacterial community, we hypothesized that feral pig removal would result in a less diverse soil bacterial community, likely due to the loss of nutrient inputs associated with feral pigs including urine and feces deposition, mirroring prior long-term removal work completed in grassland ecosystems^11,12^. We therefore additionally hypothesized that functional and phylogenetic diversity would decrease following feral pig removal due to the loss of groups associated with these nutrient pulses. We further hypothesized that bacterial diversity would not change over time following the extended removal of feral pigs due to the ability of soil bacterial communities to rapidly adapt to environmental changes^17,38,39^, which we surmised would outweigh characteristics of the chronosequence shown to change more slowly by prior work^18,27^. In regards to the influence of environmental factors (Table 1), we hypothesized that: (A) these variables would correlate to differences in the bacterial community, but would be less predictive of community dissimilarities than would the presence or removal of feral pigs; and (B) variables indirectly associated with feral pig removal (e.g. SBD and understory plant composition) would be more influential on changes to the bacterial community than environmental variables not associated with feral pigs (e.g. mean annual temperature (MAT) and elevation). These two hypotheses are driven by the understanding that feral pigs are highly influential on community composition in this system^16,18^.

## Results

Across all samples, eDNA extraction and Illumina MiSeq sequencing resulted in 53,140 to 81,823 raw reads per sample with qubit quantification results ranging from 42.0–85.6 ng/uL. Subsequent quality control and delineation of operational taxonomic units (OTUs) with QIIME 2 resulted in 8,503 total OTUs being included in our analyses (Appendix 1). PICRUSt analysis further organized these OTUs into 328 functional groups (Appendix 2).

**Figure 1:**
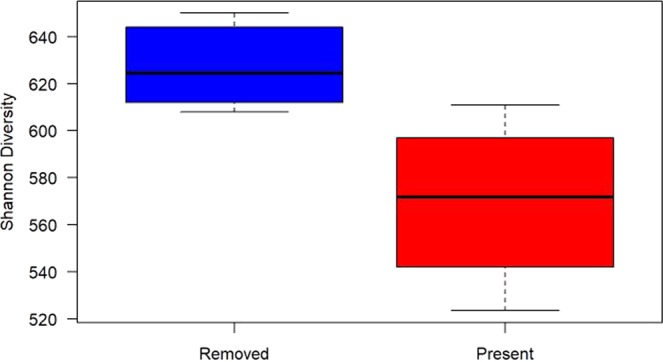
Shannon diversity in comparison to the removal/presence of feral pigs. Sites were categorized into those with feral pigs removed (in blue) and sites with feral pigs present (in red). The mean Shannon diversity score for these sites indicates that soil bacterial communities are more diverse following the removal of feral pigs (*n* = 9, *Σ*^2^ = 4.86, *p* = 0.03).

**Figure 2:**
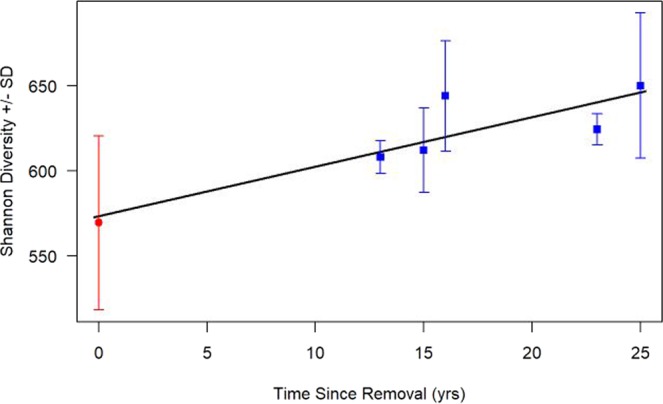
Changes in Shannon diversity score following time since feral pig removal. Following the removal of feral pigs, we see a positive linear association in the Shannon diversity of soil bacterial communities (*n* = 6, *F* = 14.81, *p* = 0.02, *r*^2^ = 0.73). Along the left side of the figure at time = 0, the red circle represents the mean of the four sites with feral pigs still present all of which do not have removal. The remaining five points symbolized with blue boxes represent each of the five plots with feral pigs removed. Error bars represent standard deviation.

Our initial assessment of the effects of environmental variables on bacterial community dissimilarity indicated that mean annual temperature (MAT), elevation, SBD, soil porosity, soil potassium (K), soil calcium (Ca), and the sum of stem density of large trees, small trees and shrubs, and tree ferns were all potential predictors of variation among bacterial communities at each of the nine sites. Following this assessment, comparing metrics of biodiversity (Shannon biodiversity index, rarified richness, Faith’s phylogenetic distance, functional Shannon biodiversity index, and functional rarefied richness) with the study site variables deemed as useful predictors led to three primary results. First, only the removal of feral pigs was associated with changes to overall bacterial community biodiversity. Specifically, the removal of feral pigs resulted in higher Shannon diversity scores (*n* = 9, *Σ*^2^ = 4.86, *p* = 0.03; Fig. 1), which was mirrored by results showing an increase in Shannon diversity scores over time following removal (*n* = 6, *F* = 14.81, *p* = 0.02, *r*^2^ = 0.73; Fig. 2). However, the removal of feral pigs did not directly result in any changes to either the functional (*n* = 9, *Σ*^2^ = 2.16, *p* = 0.14) or phylogenetic composition of bacterial communities (*n* = 9, *Σ*^2^ = 0.54, *p* = 0.46).

Instead, our second primary result indicated that environmental characteristics were the principle variables explaining variation in functional community structure across our study sites. Individually, Shannon diversity scores of functional groups were specifically associated with SBD (*n* = 9, *F* = 18.37, *p* = 0.004, *r*^2^ = 0.68), soil porosity (*n* = 9, *F* = 17.55, *p* = 0.004, *r*^2^ = 0.67), soil K (*n* = 9, *F* = 13.80, *p* = 0.008, *r*^2^ = 0.62), and soil Ca (*n* = 9, *F* = 6.13, *p* = 0.04, *r*^2^ = 0.39). Comparatively, the functional richness of the bacterial community at each site was associated with MAT (*n* = 9, *F* = 32.82, *p* < 0.001, *r*^2^ = 0.80), elevation (*n* = 9, *F* = 37.05, *p* < 0.001, *r*^2^ = 0.82), SBD (*n* = 9, *F* = 8.70, *p* = 0.02, *r*^2^ = 0.49), soil porosity (*n* = 9, *F* = 8.58, *p* = 0.02, *r*^2^ = 0.49), soil K (*n* = 9, *F* = 37.37, *p* < 0.001, *r*^2^ = 0.82), and soil Ca (*n* = 9, *F* = 29.33, *p* < 0.001, *r*^2^ = 0.78).

**Figure 3:**
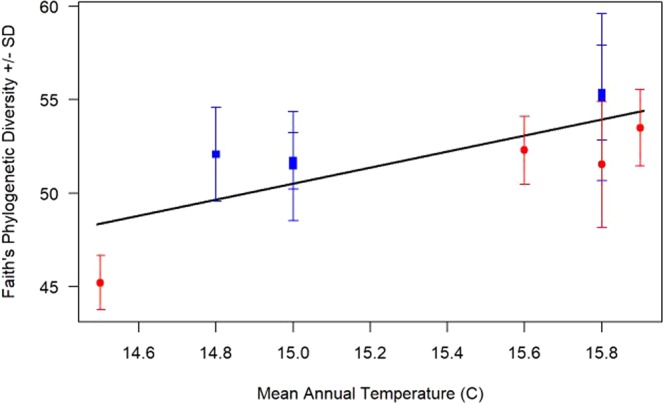
Correlating mean annual temperature (MAT) to Faith’s phylogenetic diversity. Faith’s phylogenetic diversity of the bacterial communities (*n* = 9, *F* = 10.00, *p* = 0.02, *r*^2^ = 0.53) was positively and linearly associated with MAT. Red circles represent sites with pigs present and blue boxes are sites where pigs have been removed. Error bars signify standard deviation.

Our third primary result indicated that only two environmental variables were associated with changes in the phylogenetic diversity of the bacterial communities at each site: MAT (*n* = 9, *F* = 10.00, *p* = 0.02, *r*^2^ = 0.53; Fig. 3) and elevation (*n* = 9, *F* = 8.10, *p* = 0.02, *r*^2^ = 0.47). In these cases, small changes in MAT (~1.5 °C) were associated with increased Faith’s phylogenetic diversity scores while increases in elevation (~200 m) were associated with decreased diversity scores. Importantly, MAT and elevation are linearly and inversely correlated in this system. The remaining environmental variables tested (including geographic distance between sites using principle coordinates of neighbor matrices (PCNM), size of removal units, mean annual precipitation (MAP), ground covered by litter, ground covered by bryophytes, count of terrestrial seedlings, water-filled pore space, soil magnesium (Mg), and the sum of basal area of large trees, small trees and shrubs, and tree ferns) were not significantly associated with any biodiversity metrics.

**Figure 4:**
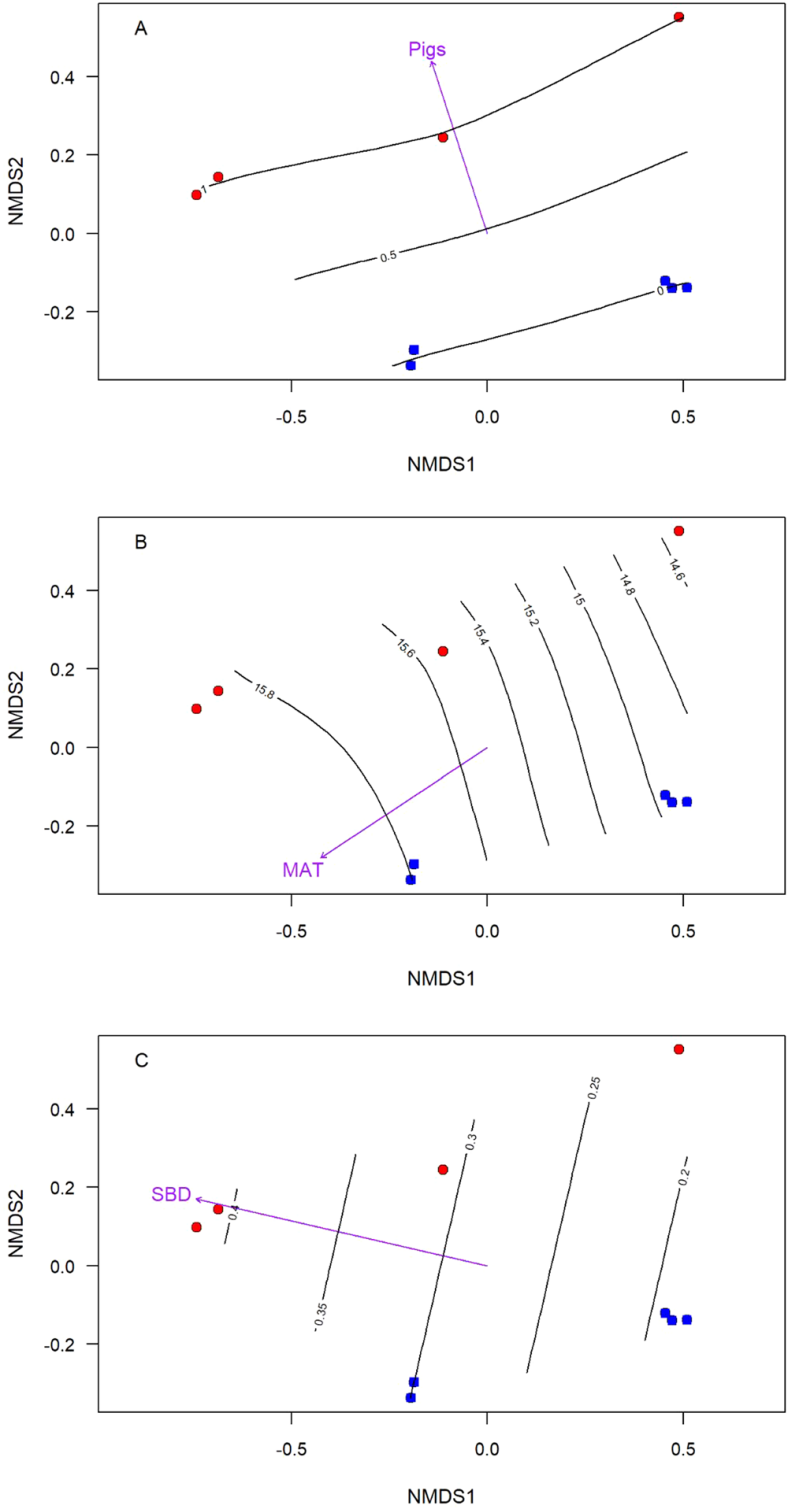
(**A–C**) Explaining bacterial community dissimilarity. Dissimilarities among bacterial communities were scored along two axes established by differences in the frequencies of individual operational taxonomic units (OTUs) at each site and placed into a dissimilarity index, displayed here. Each of the two non-metric multidimensional scaling (NMDS) axes describes dissimilarity between communities along an arbitrary scale; these two scales are the x and y axes of this figure. Within the figure, the circles represent the bacterial community at the individual sites in our study; blue circles have feral pigs present and red circles represent sites with feral pigs removed. The location of sites in the scaling space is the same across all three figures. In Fig. 4A, the purple sub-axis labeled “Pigs” represents the presence/absence of feral pigs at each site, and the horizontal lines within the figure show the scale of this axis where 1 represents sites with pigs present and 0 represents sites with pigs absent. This presence/absence is a good predictor of the dissimilarity among the bacterial communities (*p* = 0.004, *r*^2^ = 0.99). In Fig. 4B, the purple sub-axis labeled “MAT” represents mean annual temperature (MAT) at each site. The vertical lines within the figure scale the MAT from 14.5–15.9 °C. MAT was a significant predictor of dissimilarity among the bacterial communities (*p* = 0.003 *r*^2^ = 0.93). In Fig. 4C, the purple sub-axis labeled “SBD” represents soil bulk density (SBD) at each site. The vertical lines within the figure scale the SBD from 0.19–0.53 g/cm^3^. SBD was a significant predictor of dissimilarity among the bacterial communities (*p* = 0.01, *r*^2^ = 0.79).

The results of our non-metric multidimensional scaling (NMDS) analysis indicated that the presence or removal of feral pigs (*p* = 0.01, *r*^2^ = 0.99; Fig. 4A), MAT (*p* = 0.003, *r*^2^ = 0.93; Fig. 4B), and SBD (*p* = 0.01, *r*^2^ = 0.79; Fig. 4C) were the only significant predictors of bacterial community dissimilarity between sites in the simplified model. None of the other variables associated with changes in biodiversity metrics (time since feral pig removal, elevation, soil porosity, soil K, soil Ca, and the sum of stem density of large trees, small trees and shrubs, and tree ferns) were significant predictors of bacterial community dissimilarity.

## Discussion

We originally hypothesized that the diversity of soil bacterial communities would decrease following the removal of feral pigs and would not vary over time following the removal of feral pigs, with functional and phylogenetic diversity following a similar pattern. Additionally, we hypothesized that the removal of feral pigs as a direct effect would better predict dissimilarities among soil bacterial communities than the indirect and unrelated effects of individual environmental variables. Our results, however, did not support these hypotheses. Instead, the diversity of soil bacterial communities was significantly higher in sites where feral pigs had been removed compared to unfenced sites, and diversity increased positively and linearly with time since the removal of feral pigs. Further, neither functional nor phylogenetic metrics of diversity showed overall increases associated with time since removal. Rather, functional diversity and phylogenetic diversity remained relatively constant in response to feral pig removal, as well as over time following removal. Finally, both MAT and SBD were also identified as useful predictors of bacterial community dissimilarity in addition to the removal of feral pigs. Changes in SBD have been previously associated with the removal of feral pigs in our study system^27^ and can be viewed as an indirect effect of feral pig removal, and differences associated with MAT are unrelated to effects by feral pigs.

In consideration of the direct effects of feral pigs on the soil bacterial community, prior research has indicated that feral pig activity (including rooting, trampling, etc.) leads to positive increases in abundance and activity of soil microbes^6,35,36^. Further, the long-term removal of ungulates from grassland systems resulted in decreased soil bacterial diversity^11,12^ leading us to hypothesize that soil bacterial diversity would decrease following the removal of feral pigs. However, the results of our study directly conflict with these prior results despite similar trends of above- and belowground recovery observed in our study system and the system examined by Wang, *et al*.^12^. Specifically, in both systems, plant cover, plant diversity, and soil nutrient levels all increased following the exclusion of ungulates. A likely explanation for this dichotomy is the stark contrast between the two study systems being a grassland and a tropical wet forest. However, this does not mechanistically explain the differences we observed, and our study is limited in its ability to discern these differences.

While we are unable to explain a causative agent for the difference observed between our study and Wang, *et al*.^12^, we can speak to the some of the indirect effects of ungulate removal on the bacterial community in our own system. In our study, we identified SBD as a useful predictor of dissimilarity among soil bacterial communities, with lower values of SBD correlating to increased bacterial diversity. Additionally, increased levels of soil porosity, K, and Ca were all also associated with increased levels of functional diversity. In order to understand these effects as indirectly related to feral pigs, it is important to note that prior research in our study system has indicated that feral pig removal resulted in a significant decrease in SBD as well as significant increases in soil porosity, K, and Ca^27^. As such, changes to the soil bacterial community associated with these variables can largely be attributed to indirect effects of feral pig removal. These results are supported by prior work indicating that soil characteristics can explain changes to the soil microbial community^2–4^, but in our study, changes associated with soil characteristics can be indirectly attributed to feral pigs.

Our results also indicate that changes in MAT and elevation both correlate to changes in the functional and phylogenetic diversity of soil bacterial communities. Naturally, neither of these variables are affected by feral pigs, and they are inversely and linearly correlated to one another. In our analysis of these variables, MAT was a good predictor of dissimilarity among bacterial communities. This result was exemplified by the increased functional and phylogenetic diversity at warmer sites, despite small changes in MAT (~1.5 °C), thereby suggesting that, while feral pig removal does result in changes to the soil bacterial diversity, overall community structure is more sensitive to fine-scale differences in environmental variables (i.e., MAT). This result aligns with previous work showing that soil microbial communities are highly sensitive to environmental factors at small scales^3,9^. Specifically, studies conducted worldwide have indicated that temperature plays a critical role in the diversity and composition of soil microbial communities^40,41^ and has been supported by recent work suggesting microbes follow Humboldt’s patterns of tropical plant species richness, decreasing in correlation to decreased temperature and increased elevation across a much larger gradient than used in our study^42^. However, it is important to note that our results contradict those previously reported by another field study conducted in Hawai’i suggesting that soil microbial communities are only minimally affected by changes in MAT^2^. This contradiction may indicate that some other variable, such as soil nitrogen, may be influenced by MAT^43^ in our system resulting in changes to the soil bacterial community across a small gradient that were not captured by our study.

The results of this study provide important information for understanding changes over time in soil bacterial communities following the removal of nonnative ungulates, an increasingly common management approach globally. In this study, we observed a linear increase in soil bacterial diversity over time following feral pig removal that did not directly correlate to any increases in functional or phylogenetic diversity. This result indicates increases in functional overlap over time, which for some communities can increase ecosystem stability^43^. In our study area, the soil attributes we observed influencing these changes in functional diversity (including SBD, soil porosity, soil K, and soil Ca) were previously identified as changing following the removal of feral pigs^27^. As such, we can attribute the increase in functional overlap indirectly to the removal of feral pigs. Comparatively, phylogenetic differences between bacterial communities were only associated with variables that are not influenced by feral pigs (i.e. MAT and elevation) leading us to conclude that the phylogenetic structure of bacterial communities at our study site is not influenced, directly nor indirectly, by feral pigs. In the future, it is likely that a more in-depth functional metagenomic analysis could improve our understanding of this system.

## Methods

### Study site

This study utilized a chronosequence of feral pig removals on the Island of Hawai’i initially characterized by Cole and Litton^18^. The experimental design consists of five pairs of sites arrayed across feral pig removal units located on the eastern side of Mauna Loa Volcano in the Ola’a Unit of Hawai’i Volcanoes National Park and the adjacent Pu’u Maka’ala Natural Area Reserve. These pairs include four sites with feral pigs present and five sites with feral pigs removed (with one pig present site serving as the pair for two pig removal sites) at intervals ranging from ~13 to ~25 years prior to our study^18^. One limitation of this study is the lack of spatial replicates, and our results should therefore be interpreted with appropriate caution. However, this system is the best available for analyzing differences in wet forest ecosystems because there are no longer any naturally occurring pig-free areas within these habitats in Hawai’i, and the cost of constructing these units is high due to their remote location and rugged terrain. Combined, these factors inherently limit the study of these processes in regards to feral pigs. Further, these removal units range in size from 117–1,024 ha providing enough geographic space for spatial replicates, but doing so would result in the pseudoreplication of data.

All study sites occur on 2,000–10,000 year-old tephra-derived andisols that are characterized by deep, moderately well-drained soils from basic volcanic ash deposited over basic lava. These soils stem from two closely related soil series: Puaulu (medial over ashy, aniso, ferrihydritic over amorphic, isothermic Aquic Hapludands) and Eheuiki (medial, ferrihydritic, isothermic Typic Hydrudands) with slopes of 2–5%^18,27,44^. Sites range in elevation from 1,140–1,370 m above sea level, with MAT inversely correlating to elevation and covering a range of 14.4–15.9 °C. MAP is between 2,910–3,985 mm at the sites with no distinct seasonality^45^. Vegetation is characterized by large areas of canopy-intact native tropical montane wet forests classified as *Metrosideros polymorpha*/*Cibotium* spp.^46^. Feral pig densities have been estimated at 0.6–16.3 feral pigs/km^2^ at these sites^47^, and excluding recreational hunting, forests surrounding the exclosures have never been managed for feral pigs^18,27^. Significant prior work has been conducted in these sites and the information from these studies, which we have utilized for the sake of our own analyses, is summarized in Table 1; however, this is not an exhaustive list of all variables that could possibly be studied within this ecosystem.

### Soil collection

During May of 2017, three soil samples were collected from unique plots within each of the nine study sites described above for a total of 27 samples. In order to control for canopy cover and associated aboveground characteristics in the local environment, the center of each plot was placed within 1 m of a mature tree fern (*Cibotium* spp.). Each sample was collected by gathering eight subsamples from 1 and 3 m intervals in the four cardinal directions from each plot’s center. Leaf litter was cleared to expose Oa- and A-horizon soils, and a clean spatula, latex gloves, and Falcon tube were used to collect each soil subsample (<12 mL in volume) from the top 1–2 cm of the soil from each of the eight subsample points. Once the subsamples were collected and sealed into a single Falcon tube representing the entire sample, the sample was shaken by hand to homogenize the soil and placed in a cooler for ~3–7 hours until it could be transferred to a −20.0 °C freezer for longer-term storage until analysis.

### eDNA sequencing and analysis

To assess the soil bacterial community in each subplot, eDNA was extracted from the soil samples using MP Biomedicals’ FastDNA SPIN Kit for Soil (MP Biomedicals, Santa Anna, California, USA). Manufacturer provided protocols were followed, including extended centrifugation at 14,000 rpm to remove excessive debris and an extra 5 minutes of incubation at 55 °C to increase eDNA yields. The standard Illumina 16S Metagenomic Sequencing Library Preparation workflow was used to prepare samples for sequencing the 464 bp variable V3 and V4 regions of the 16S rRNA gene^48^ using the primer pairs forward S-D-Bact-0341-b-S-17 (5′-CCTACGGGNGGCWGCAG-3′) and reverse S-D-Bact-0785-a-A-21 (5′-GACTACHVGGGTATCTAATCC-3′)^49^. These primers were concatenated to standard Illumina adapters, annealed to eDNA sample extracts at 95 °C for 3 minutes, and amplified using KAPA HiFi HotStart ReadyMix for 25 cycles at 95 °C for 30 seconds, 55 °C for 30 seconds, and 72 °C for 30 seconds and finally held at 72 °C for 5 minutes^48^. Amplified products were purified twice using AMPure XP magnetic beads (Beckman Coulter, Brea, California, USA) and quantified using Biotium’s AccuBlue High Sensitivity dsDNA Solution Kit on a Qubit 3.0 fluorometer per manufacturer guidelines (Biotium, Fremont, California, USA). Libraries were combined at equimolar concentration and sequenced at the University of Hawai’i at Mānoa Advanced Studies in Genomics, Proteomics, and Bioinformatics (ASGPB) genomics core facility on a 300 bp paired-end Illumina MiSeq platform. All samples were multiplexed using the Illumina NextEra XT index kit. Negative controls did not result in any sequences after processing and were thereafter removed from our analyses. Raw sequence reads were deposited in MG-RAST (Project # 87547).

Molecular sequences were processed using the QIIME 2 bioinformatics platform version 2018.4 within a Virtual Box Core^50^. Initial sequences were demultiplexed and truncated at the 10^th^ bp from the left for both the forward and reverse reads. From the right, the sequences were truncated from the 290^th^ bp on the forward read and from the 250^th^ bp on the reverse read. Divisive Amplicon Denoising Algorithm (DADA2) was then used to merge paired reads, filter by sequence quality, denoise, and create a sample × OTU table, and remove chimeras^51^. OTUs were then taxonomically assigned using the Naïve Bayes Classifier trained on the Greengenes 13_8 99% OTU database. OTUs occurring <10 times across all 27 samples were removed. The final OTU table was rarefied to 8,503 sequences and used for all subsequent analyses (Appendix 1). Secondarily, OTUs were assigned to functional groups using Phylogenetic Investigation of Communities by Reconstruction of Unobserved States (PICRUSt) bioinformatics software^51^. This step was completed with an open reference search within the QIIME 2 platform of the Greengenes 13_5 database. The resultant file listed the predicted functional groups based-off specific 16S rRNA OTUs^52^ (Appendix 2). These functional groups are established by placing each OTU into a functional category determined by their phylogeny and related information in the Greengenes database. QIIME 2 was then used to obtain biodiversity scores: Shannon biodiversity index, rarified richness, and Faith’s phylogenetic distance as well as Shannon biodiversity index and rarefied richness describing predicted functional diversity.

### Statistical analyses

Data from each of the 27 total samples collected was averaged across the three samples from each site resulting in statistical analyses of the nine sites existing in the chronosequence (*n* = 9). Statistical analyses were then conducted using R-Studio Version 3.3.4^53^. Biodiversity scores obtained in QIIME 2 were compared against available site characteristics obtained from Cole and Litton^18^, Long, *et al*.^27^, and Giambelluca *et al*.^45^ summarized in Table 1. Due to the non-parametric nature of the data, Kruskal-Wallis rank sum tests were used to determine if any differences in biodiversity were associated with categorical site characteristics including presence/absence of feral pigs (Fig. 1) and soil series. Comparatively, continuous site characteristics (time since removal, MAP, MAT, elevation, etc.) were compared in relation to biodiversity metrics using linear regression (Figs. 2 and 3).

In addition to comparisons of biodiversity metrics, NMDS vector fitting was used to fit site characteristics and comparisons of Euclidean distance was completed using principle coordinates of neighbor matrices (PCNM). These analyses were conducted using the <vegan> package available within R^54^. For each subset, basic ordination of community structure was established at each site using linear directional gradients <envfit>. These vectors were then plotted with surface fitting for improved clarity of nonlinear vector relationships <ordisurf>. Finally, constrained correspondence analysis (CCA) was used to determine site characteristics significantly affecting community composition dissimilarities to 1,000 iterations. Permutation tests were then conducted to select the best fitting model (Fig. 4A–C).

## Supplementary information

Appendix 1

Appendix 2

## Data Availability

Raw data is identified in the manuscript as Appendices 1 & 2 and have been submitted with the manuscript. Additionally, raw genetic sequences are publicly availableas MG-Rast Project #87547.
